# When sounds come alive: animacy in the auditory sense

**DOI:** 10.3389/fpsyg.2024.1498702

**Published:** 2024-10-25

**Authors:** Stefano Gonan, Giorgio Vallortigara, Cinzia Chiandetti

**Affiliations:** ^1^Department of Life Sciences, University of Trieste, Trieste, Italy; ^2^CIMeC - Center for Mind/Brain Sciences, University of Trento, Rovereto, Italy

**Keywords:** auditory perception, animacy, voicelikeness, acoustic motion, music

## Abstract

Despite the interest in animacy perception, few studies have considered sensory modalities other than vision. However, even everyday experience suggests that the auditory sense can also contribute to the recognition of animate beings, for example through the identification of voice-like sounds or through the perception of sounds that are the by-products of locomotion. Here we review the studies that have investigated the responses of humans and other animals to different acoustic features that may indicate the presence of a living entity, with particular attention to the neurophysiological mechanisms underlying such perception. Specifically, we have identified three different auditory animacy cues in the existing literature, namely voicelikeness, consonance, and acoustic motion. While the first two characteristics are clearly exclusive to the auditory sense and indicate the presence of an animate being capable of producing vocalizations or harmonic sounds—with the adaptive value of consonance also being exploited in musical compositions in which the musician wants to convey certain meanings—acoustic movement is, on the other hand, closely linked to the perception of animacy in the visual sense, in particular to self-propelled and biological motion stimuli. The results presented here support the existence of a multifaceted auditory sense of animacy that is shared by different distantly related species and probably represents an innate predisposition, and also suggest that the mechanisms underlying the perception of living things may all be part of an integrated network involving different sensory modalities.

## 1 Introduction

Why are some sounds perceived as coming from living things and others not? Is there a sense of auditory animacy in different animal species? It is well known that animacy perception plays a crucial role in the survival of animals from an early age, enabling individuals to automatically and effortlessly locate biological cues of animate beings and then react according to the nature of the perceived entity, predator, prey, conspecific, etc. However, most research in this area has focused on the visual domain, particularly exploring the role of biological motion (Lorenzi et al., 2024), face perception (Kobylkov and Vallortigara, 2024; Kobylkov et al., 2024), and changes in speed and direction of moving objects (Di Giorgio et al., 2017; Lorenzi et al., 2017; review in Lorenzi and Vallortigara, 2021), while few researchers have addressed the possibility of an auditory counterpart to animacy perception (but see Tremoulet and Feldman, 2000).

This relative paucity of studies should not be surprising, however, given that auditory perception is characterized by a completely different psychophysical nature compared to the visual modality. As a result, the features that might constitute an animacy cue in the auditory sense are not necessarily the same as those that have been identified in visual research. When we think about how animals, relying solely on their sense of hearing, are able to discriminate between living and non-living entities, the first feature that comes to mind is something unique to the auditory sense: the voice. Indeed, authors such as Patterson (2014) identify in the pulse-resonance structure of animal voices one of the key features at the basis of living organisms’ recognition through sound.

It follows that all those sounds that are sufficiently similar in their acoustical structure to that of the voice—the so-called *voicelikeness*—should be able to elicit neurophysiological and behavioral responses similar to those of the voice (Broze, 2013). This was used by occultists at the turn of the 19th and 20th centuries, who argued for an ability allegedly demonstrated by certain people to perceive human voices in the noise produced by mechanical-electronic devices such as the radio, a phenomenon that became known as Electronic Voice Phenomena (Leary and Butler, 2015). They were actually documenting the auditory equivalent of pareidolia (a well-known phenomenon in vision), i.e., misperceiving or misinterpreting random noise or ambiguous acoustic stimuli as meaningful sounds, such as words or even sentences (Blom, 2015). This perceptual illusion suggests that the human species has a hypersensitivity to searching for and identifying voices within the auditory scene in which one is immersed, similar to what happens in the visual modality when faces are perceived in inanimate objects (Liu et al., 2014) also by non-human primates (Taubert et al., 2017). From a neuroanatomical perspective, the processing of voices of both conspecific and heterospecific individuals is supported by a dedicated extensive network of cortical areas (Broze, 2013).

In addition, another acoustic feature at the basis of the recognition of animate entities is consonance, i.e., sound frequencies that, when combined, produce the perception of a single sound and are therefore considered pleasant (Trulla et al., 2018). Because this feature is prominent in all acoustic events characterized by a well-defined and clear harmonic structure—such as the vocalizations of many species of songbirds and human speech—it is considered a hallmark of sounds produced by animate beings, which would explain the attraction that consonance exerts from an early age in distantly related taxa such as humans, domestic chicks and chimpanzees (Chiandetti and Vallortigara, 2011).

Finally, the perception of animacy in the auditory sense also occurs because of another acoustic feature that is closely related to one of the most important visual cues to animacy, i.e., locomotory movement. When animals move through space, they frequently simultaneously produce characteristic sounds—just think of the buzzing of a flying insect—defined as motion-induced sounds (Clark, 2016). These are involuntary acoustic events generated by the movement of anatomical parts of the body that are not specialized in producing communicative sounds, so that their conveyed information—i.e. the presence of a moving animal—is exploited by listeners rather than being transmitted directly and voluntarily (Clark, 2016). Neuroscientific studies have also demonstrated the existence of brain areas dedicated to the detection of acoustic motion, similar to those devoted to motion perception in other sensory modalities, such as vision (Wagner et al., 1997).

This work offers one of the first reviews in the field of auditory animacy perception and aims to fill this gap by systematizing the existing knowledge into a coherent description, identifying the main lines of research and proposing a global interpretation of the results obtained.

## 2 Voicelikeness

In vertebrates, voice production and perception are phenomena with a long evolutionary history. Voice generation mechanisms are the output of a system, the vocal tract, whose functional and neural control components are highly conserved across species (Newman, 2010; Fitch and Hauser, 2003). Furthermore, the ability to perceive conspecifics’ voice is encoded in species-specific auditory nuclei in the auditory forebrain of birds (Louder et al., 2019) and, in the mammalian brain, regions such as the superior temporal plane and auditory neurons in the ventrolateral prefrontal cortex are involved in all studied primate species (Petkov et al., 2008; Romanski and Averbeck, 2009), with the anterior superior temporal sulcus specialized in humans (Belin et al., 2000; Fecteau et al., 2004). Moreover, the ability to recognize and approach the voice of a conspecific, especially if it is the mother’s, is an ability shown early in life in many avian species, such as domestic chicks (Gottlieb, 1965; Fält, 1981; Bolhuis and van Kampen, 1991), pekin ducklings (Gottlieb, 1965), wood ducklings (Gottlieb, 1965), willow grouses (Allen, 1977), Japanese quails (Park and Balaban, 1991) and bobwhite quails (Barrow Heaton et al., 1978), which prefer the voice of their own species to that of another taxon or noise. As shown by Long et al. (2001), species-specific neural development underlies auditory preferences in taxa such as the domestic chicken and the Japanese quail: specifically, the authors transplanted developing neural tubes from embryonic quails to embryonic chickens and then tested the auditory preferences of the chimeric domestic chicks, finding that they began to prefer quail vocalizations.

However, voice-sensitive brain areas also recognize specific characteristics of the voice—such as fundamental frequency, call length and harmonic and phase-coupling content—regardless of the species. These neural correlates include the middle portions of the left and right superior temporal gyri, the right posterior superior temporal gyrus, the left Heschl’s gyrus and left planum temporale in humans (Lewis et al., 2005; Bálint et al., 2023) and the mid and caudal ectosylvian gyri in dogs (Bálint et al., 2023). It can thus be posited that there exists an ancient neural predisposition to perceive sounds that exhibit characteristics that are analogous to those observed in acoustic events produced by vocal folds vibrations, a feature called *voicelikeness* (Schubert and Wolfe, 2016; Bálint et al., 2023). Consequently, auditory stimuli characterized by vocal similarity to certain innately encoded acoustic features will elicit a preference/approaching response. For example, one- and three-day-old domestic chicks run faster toward pure tones or tapping sounds when their frequency (Fischer, 1972), duration (Fischer, 1972), intensity (Fischer and Gilman, 1969), and rate (Fischer, 1972) are similar to those of the ideal maternal attraction call (Collias and Joos, 1953; Collias, 1987; Kent, 1993). In addition, sounds that emphasize a particular fundamental feature may be perceived as a superstimulus, and then be preferred over the original natural vocalization, as is the case with three-day-old chicks that run faster toward stimuli with a higher rate than natural mother’s clucks (De Tommaso et al., 2019).

Most importantly, as demonstrated by Gilbert Gottlieb and colleagues in different auditory vs. visual choice experiments studying imprinting in domestic chicks (Gottlieb and Simner, 1969), pekin (Gottlieb and Klopfer, 1962; Klopfer and Gottlieb, 1962), mallard (Gottlieb, 1968) and wood ducklings (Gottlieb, 1968), the magnitude of attractiveness of vocal stimuli as animacy cues exceeds that of visual stimuli, showing that—at least in the first days of life—the auditory recognition prevails, as ducklings prefer to follow a concealed moving loudspeaker broadcasting the species-specific maternal call instead of a silent moving visual replica of the hen, while domestic chicks are more attracted to an even simpler stimulus as an auditory flickering than to its visual version. Similarly, in the early stages of development, bobwhite quail chicks primarily rely on auditory cues for species identification and filial behavior, but as they mature, visual cues are integrated, with auditory stimuli remaining dominant (Lickliter and Virkar, 1989). Overall, these results support the cue hierarchy hypothesis proposed by Johnston and Gottlieb (1985), according to which sensory systems are organized hierarchically in early development, with the brain giving greater priority to auditory information as the auditory system matures faster than the visual system (Lickliter and Virkar, 1989).

## 3 Consonance

Analyses of the human voice have shown that when frequency and intensity values intersect, peaks corresponding to consonant melodic intervals emerge, suggesting that consonant intervals may represent the default state of human intonation (Schwartz et al., 2003). This phenomenon aligns with a distinctive characteristic of biological vocalizations: the presence of a well-defined harmonic structure, consisting of a fundamental frequency and harmonic overtones—a feature also shared by the sounds produced by musical instruments. This aspect was first noted in the 18th century by the French music theorist and composer Jean-Philippe Rameau, who observed that there was a connection between tonal sounds—often produced by living beings—and harmonic structure, particularly consonance (Christensen, 2004). Consonance is defined as the combination of two or more sound frequencies played simultaneously or consecutively that the brain perceives as stable, predictable and qualitatively pleasing. Conversely, dissonance results from the interaction of frequencies that when combined result as unstable, thus creating a perception of roughness or harshness in the auditory system.

This intuition has been confirmed by a number of studies of the vocalizations of different oscine species, such as the musician wren and the great tit, whose songs are characterized by a heavy use of consonant notes (Doolittle and Brumm, 2012; Richner, 2016), or, like the hermit thrush, which arranges its songs according to an overtone structure (Doolittle et al., 2014). Furthermore, in great tits, there is also a relationship between male fitness and the accuracy with which they produce consonant notes in their songs, showing that females prefer sounds characterized by stability and predictability (Richner, 2016). However, it is not even necessary for an animal to sing in order to “speak” consonant, since domestic chicks, for instance, emit perfect consonances across all types of calls, highlighting that consonant sounds are inherently present in animal communication (Maldarelli et al., 2024). Not only that, but the tone of our species’ voice in dyadic interactions was shown preliminarily to be consonant when there is agreement between the speakers, and dissonant when disagreement arises (Okada et al., 2012).

Taken together, these results suggest a fundamental similarity between the harmonic structure of periodic sounds, whether the sound of the voice or that of musical instruments, thus making consonance a prominent aspect of vocalizations emitted by animate beings (Bowling and Purves, 2015; Wagner and Hoeschele, 2022). This conclusion is further supported by the evidence that, in studies employing a spontaneous preference paradigm, consonant sounds are favored over dissonant ones in newborns of distantly related species, including domestic chickens (Chiandetti and Vallortigara, 2011), chimpanzees (Sugimoto et al., 2010) and humans (Masataka, 2006; Perani et al., 2010). This finding corroborates the idea that consonance is employed as a distinctive auditory cue for the presence of an animate object (Chiandetti and Vallortigara, 2011; Chiandetti, 2016; Vallortigara, 2021), as opposed to an inanimate one. Moreover, it suggests that this discriminative capacity may be a shared trait among species and may constitute an acoustic unlearned predisposition before experience, culture and training shape our preferences (Lahdelma and Eerola, 2020; Prete et al., 2020; Lahdelma et al., 2022).

## 4 Acoustic motion

The world is full of things that move and produce sound as a by-product. Some of them are alive—and the acoustic events produced are then called motion-induced sounds—and some of them are not, like leaves rustling, rocks tumbling or ocean waves. So, how does the auditory system discriminate between sounds produced by moving biological entities and those produced by moving objects? The first study to attempt to answer this question is that of Bidet-Caulet et al. (2005), in which the authors used fMRI technique to investigate in humans the neural correlates of auditory biological motion, more precisely the perception of footsteps. The results showed that the superior temporal sulcus was mainly involved in the processing of human motion sounds, irrespective of the sensory modality. A few years later, Cottrell and Campbell (2014) investigated auditory sensitivity to footsteps compared to non-biological impact sounds, such as a bouncing ball or drumbeats. Contrary to expectations, they found no increased sensitivity to biological motion compared to non-biological sounds, a finding that seems to indicate a difference between sensory modalities, with the visual domain being more fine-tuned when compared to the auditory domain. However, despite this discrepancy, both studies seem to converge on one aspect, namely the importance of temporal cues in the detection of human motion, especially if related to footsteps, a sound with high biological value (Bidet-Caulet et al., 2005; Cottrell and Campbell, 2014; Larsson, 2014).

The role of timing in the perception of auditory animacy was later taken up in the field of musical cognition, where there is a debate about the characteristics impacting the “aliveness” of a musical piece performance, an issue that, as some authors have noted (Blust et al., 2016), overlaps with that of auditory animacy. In particular, the bridge linking music to animacy is *rubato*, a performance technique used to convey greater expressiveness consisting of slight changes in the timing of a musical piece by slowing it down or speeding it up, giving also an acoustic motion sensation. In their 2015 paper, Blust and colleagues investigated the role of rubato in making music sound animated, asking participants to rate on a Likert scale the perceived animacy of computerized and human performances, both varying in rubato level. Results showed that both fixed and excessive timing variations led to a decrease in perceived animacy, while minimal levels of rubato resulted in significantly higher ratings of animacy (Blust et al., 2016).

The work of Nielsen et al. (2015), instead, made a significant contribution to the advancement of the field by investigating the effects of cues such as changes in the speed and direction of acoustic motion on the perception of animacy in humans. To this end, they designed a study that represents the auditory analog of the previously conducted research in the visual domain by Tremoulet and Feldman (2000). This was done by using binaural spatialisation to generate acoustic stimuli that mimicked the motion in three-dimensional space of a synthesized mosquito sound, varying in speed and direction—thus creating the impression of a living entity—or keeping the values constant in both dimensions. Participants were then asked to rate on a Likert scale their confidence that the perceived sound was lifelike or not, showing a significant difference between changes in speed and no changes, but unexpectedly also between changes in speed and changes in direction, suggesting the existence of a hierarchical organization of animacy cues with respect to acoustic motion. No study has yet addressed whether it is possible, in the acoustic domain, to distinguish causal interactions between inanimate and animate objects, as has been observed in the visual domain early in ontogenesis (Kominsky et al., 2022).

Finally, how do animals perceive acoustic motion? Even if animacy-related data are still absent, it is known that brain structures like the inferior colliculus are at the basis of the detection of acoustic motion direction, a process that is then refined in sensitivity through GABAergic inhibition, while motion information is then further processed by higher regions such as the auditory cortex (Wagner et al., 1997). Some animal taxa specialized in hunting using auditory cues even possess auditory space maps that integrate and help localize motion information (Wagner et al., 1997). (For a comprehensive review on this topic, see Carlile and Leung, 2016).

## 5 Discussion

This review has provided a full description of the area of auditory animacy, stressing the multifaceted nature of the acoustic mechanisms at the basis of animate beings’ perception, going from voice features to sounds emitted involuntarily during biological motion. As outlined here, parallels between auditory and visual animacy cues are striking, with the voice being the acoustic analog of the face, and voicelikeness—resulting from the abstraction of vocal fundamental features—leading to a sensitivity comparable to that for face-like stimuli, causing the occurrence of phenomena such as auditory pareidolia, similar to face pareidolia. While the link between biological and acoustic movement is evident and very close, consonance as a feature of vocal prosody may instead fulfill a role akin to that of eyes and gaze, which are key characteristics in face and social perception.

Future research should consider whether face perception offers further analogies, such as in upside-down processing and backward speech, a domain where potential influences of rhythm, stress, and intonation can be examined (Toro et al., 2005). Studies should also address the potential effects of high-level cognition in modulating animacy perception—as done by Kim and Schachner (2021) who linked causal reasoning to animacy perception in music—and explore new potential cues, such as affective prosody (Zimmermann et al., 2013), or auditory regularities like fractal structure in vocal emissions (Jermyn et al., 2023). Finally, more research should investigate the cross modality of perceptual animacy, which may be part of a larger integrated system of social perception that includes different sensory modalities such as vision and hearing, and maybe even other unexplored domains, such as touch or olfaction.
